# Urinary protein and renal prognosis in idiopathic membranous nephropathy: a multicenter retrospective cohort study in Japan

**DOI:** 10.1080/0886022X.2018.1487864

**Published:** 2018-07-27

**Authors:** Makoto Yamaguchi, Masahiko Ando, Takayuki Katsuno, Naotake Tsuboi, Shoichi Maruyama

**Affiliations:** a Municipal Yokkaichi Hospital, Yokkaichi, Japan;; b Center for Advanced Medicine and Clinical Research, Nagoya University Hospital, Nagoya, Japan;; c Department of Nephrology, Nagoya University Graduate School of Medicine, Nagoya, Japan

**Keywords:** Idiopathic membranous nephropathy, urinary protein, renal prognosis, remission

## Abstract

**Background:** Several studies have revealed a relationship between proteinuria and renal prognosis in idiopathic membranous nephropathy (IMN). The benefit of achieving subnephrotic proteinuria (<3.5 g/day), however, has not been well described.

**Methods:** This multicenter, retrospective cohort study included 171 patients with IMN from 10 nephrology centers in Japan. The relationship between urinary protein over time and a decrease of 30% in estimated glomerular filtration rate (eGFR) was assessed using time-dependent multivariate Cox regression models adjusted for clinically relevant factors.

**Results:** During the observation period (median, 37 months; interquartile range, 16–71 months), 37 (21.6%) patients developed a 30% decline in eGFR, and 2 (1.2%) progressed to end-stage renal disease. Time-dependent multivariate Cox regression models revealed that lower proteinuria over time were significantly associated with a lower risk for a decrease of 30% in eGFR (primary outcome), adjusted for clinically relevant factors. Complete remission (adjusted hazard ratio [HR], 0.005 [95%CI, 0.0–0.09], *p* < .001), incomplete remission with <1.0 g/day of urine protein (ICR I) (adjusted HR, 0.01 [95%CI, 0.001–0.20], *p* = .002), and 1.0 to 3.5 g/day (ICR II) (adjusted HR, 0.12 [95%CI, 0.02–0.64], *p* = .013) were significantly associated with avoiding a 30% decrease in eGFR, compared to that at no remission.

**Conclusions:** Attaining lower proteinuria predicts good renal survival in Japanese patients with IMN. This study quantifies the impact of proteinuria reduction in IMN and the clinical relevance of achieving subnephrotic proteinuria in IMN as a valuable prognostic indicator for both the clinician and patient.

## Introduction

Idiopathic membranous nephropathy (IMN) is common in adult nephrotic syndrome [1]. Persistent proteinuria has been considered to be associated with an increased risk for kidney dysfunction; approximately 30–40% of patients with persistent high levels of proteinuria eventually progress to end-stage renal disease (ESRD) [2,3]. In two cohort studies in Canada, complete remission (CR) of nephrotic syndrome, defined as protein excretion below 0.3 g/day, predicts excellent renal survival. Partial remission, defined as protein excretion below 3.5 g/day plus a 50% or greater reduction in protein excretion from the peak value, was also independently associated with a slower decrease in renal function and a lower incidence of renal failure [4,5]. Therefore, the primary aim of treatment in IMN was to induce a lasting reduction in proteinuria. However, those studies did not include information about proteinuria over time or the use of immunosuppressive therapy during the follow-up period; therefore, the relationship between proteinuria and renal prognosis of IMN could not be adequately assessed.

Little is known about the relationship between proteinuria and kidney dysfunction in IMN in Japanese patients. Japanese patients with IMN experience a more benign course compared to patients in other countries; Japanese patients have a higher remission rate, and the incidence rate of ESRD is relatively lower [6–10]. Additionally, the median age of patients included in our previous study [8,9] was higher than that of middle-aged patients in cohort studies from other countries [2–5]. Thus, it is unclear whether findings from studies in other countries are generalizable to patients in Japan.

The first aim of the present study was to assess the relationship between proteinuria over time and renal prognosis in Japanese IMN patients on the basis of a 30% decline in estimated glomerular filtration rate (eGFR), which has been considered the surrogate marker of ESRD in the general population [11,12], and by using time-dependent Cox regression models adjusted for clinically relevant factors. Our second aim was to determine whether a goal for reducing proteinuria to a defined maximum limit should be set.

This multicenter, observational cohort study was organized as part of the Nagoya Nephrotic Syndrome Cohort Study (N-NSCS) based at 10 major nephrology centers in Nagoya, Japan.

## Subjects and methods

### Study population and data source

The clinical data from 171 IMN patients included in the present study were derived from our previous study, N-NSCS [8,9]. The study design of N-NSCS was described in detail elsewhere [8,9]. Our study was conducted using a linkable, anonymous data set. Briefly, this cohort study included patients aged >18 years who had been diagnosed with IMN on the basis of kidney biopsy between January 2003 and December 2012 at Nagoya University, Chubu Rosai Hospital, Japanese Red Cross Nagoya Daiichi Hospital, Tsushima City Hospital, Kasugai Municipal Hospital, Nagoya Kyoritsu Hospital, Anjo Kosei Hospital, Ichinomiya Municipal Hospital, Handa City Hospital or Tosei General Hospital. Of the 272 patients in the N-NSCS study, we excluded patients with conditions generally considered to cause secondary membranous nephropathy (MN), such as exposure to drugs associated with MN, diabetes mellitus, systemic lupus erythematosus, malignancy, or any other systemic disease associated with secondary MN [10].

After exclusion of an additional 7 (3.9%) patients with missing data, 171 (62.9%) patients with IMN were enrolled and followed up with until September 2013.

No informed consent was obtained. The study protocol was approved by the ethics committees of Nagoya University, Chubu Rosai Hospital, Japanese Red Cross Nagoya Daiichi Hospital, Tsushima City Hospital, Kasugai Municipal Hospital, Nagoya Kyoritsu Hospital, Anjo Kosei Hospital, Ichinomiya Municipal Hospital, Handa City Hospital, Yokkaichi Municipal Hospital, and Tosei General Hospital.

### Measurements

During the observation period, every patient was classified into each of these four proteinuria groups at least once, depending on his or her urine protein level at the time of measurement. The time at which kidney biopsy was performed was used as baseline if a patient had not received immunosuppressive therapy or had received it only after kidney biopsy. In patients who had received immunosuppressive therapy before kidney biopsy, the time of the initiation of immunosuppressive treatment was used as baseline. Clinical characteristics taken at baseline included age, gender, body mass index (BMI = body weight [kg]/height^2^ [m^2^]), estimated glomerular filtration rate (eGFR; estimated using the equation recently generated by the Japanese Society of Nephrology: eGFR [mL/min/1.73 m^2^] = 194 × Scr^−1.094^ × Age^−0.287^ × 0.739 [if female] [13]), total cholesterol level, serum albumin level, 24-h urinary protein excretion or urinary protein-to-creatinine ratio, and use of any antihypertensive drugs, including angiotensin-converting enzyme (ACE) inhibitors or angiotensin II receptor blockers (ARB), calcium channel blockers, β-blockers, and thiazides. Details on the use of corticosteroids and/or other immunosuppressive agents during the follow-up period were also collected. The patients were generally seen every 1−2 months, and urinary protein and eGFR were tested at each visit. Nephrotic syndrome was defined as urinary protein excretion of ≥3.5 g/day (or a urinary protein/creatinine ratio of ≥3.5 g/gCr) and a serum albumin level of <3.0 mg/dL.

As previously reported [14], we classified proteinuria into four groups: CR, incomplete remission (ICR) I, ICR II, and no remission (NR). CR of proteinuria was defined as urinary protein excretion of <0.3 g/day, a urinary protein/creatinine ratio of <0.3, and/or a negative/trace result for urinary protein on a dipstick test. ICR was defined as continuing proteinuria but in a non-nephrotic range, and was divided into two grades showing <1.0 g/day of urine protein (ICR I) and 1.0 to 3.5 g/day (ICR II), respectively. NR was defined as persistent nephrotic syndrome. Relapse was defined as urinary protein excretion of ≥1.0 g/day, a urinary protein/creatinine ratio of ≥1.0, or a urinary protein dipstick result of ≥2+ on at least two occasions after achievement of CR. The rate of decline in eGFR per year (mL/min per 1.73 m^2^/year) was determined by plotting the eGFR against the observation time.

### Outcomes

The outcome of interest in the present study was a 30% decline in eGFR before ESRD. Patients were followed up with until September 2013 and censored at the time of death (if before ESRD) or as of the last eGFR measurement before September 2013.

### Statistical analysis

The cumulative probability of developing a 30% decrease in eGFR and CR was calculated using the Kaplan–Meier method and the log-rank test.

To identify predictors independently associated with outcome, baseline data such as age, sex, and serum creatinine level were examined using multivariate Cox regression models, and to evaluate the impact of urinary protein exposure on a 30% decrease in eGFR, follow-up data on urinary protein level and immunosuppressive treatment (prednisolone or prednisolone + other immunosuppressive agents) were examined using multivariate time-dependent Cox regression models. For the time-dependent models, laboratory values were updated every month, and the value of the last visit was imputed for those who had missing values. In analysis, urinary protein was divided into four groups: <0.3g/day (CR); 0.3–1g/day (ICR I); 1–3.5g/day (ICR II); and ≥3.5 g/day (NR). Least squares means ±95% confidence intervals (CI) for eGFR were calculated using a linear mixed-effect model and compared among the four proteinuria groups (CR, ICR I, ICR II, or NR), which were achieved at least one time in each patient.

The level of statistical significance was set at *p* < .05. All statistical analyses were performed using JMP version 10.0.0 (SAS Institute, Cary, NC; www.jmp.com) and SAS version 9.4 (SAS Institute, Cary, NC; www.sas.com).

## Results

### Study participants and clinical characteristics

The clinical characteristics of the 171 IMN patients are summarized in Table 1. The median age of the patients in the present study was 64 years (interquartile range, 57–70 years), median urinary protein was 4.3 (interquartile range, 2.6–7.5) g/day, median eGFR was 76 (interquartile range, 60–91) mL/min/1.73 m^2^, and 114 (66.7%) patients had nephrotic syndrome as previously defined.

**Table 1.** Clinical characteristics of patients with IMN.

	Total cohort
Number	171
Baseline characteristics	
Age (years)	64 (57–70)
Male [*n* (%)]	118 (69.0)
Body mass index (kg/m^2^)	23.1 (21.5–25.3)
Systolic blood pressure (mmHg)	130 (120–143)
Diastolic blood pressure (mmHg)	77 (70–85)
Serum creatinine (mg/dL)	0.80 (0.69–1.00)
eGFR (mL/min/1.73 m^2^)	76 (60–91)
Serum albumin (g/dL)	2.5 (2.1–3.2)
Urinary protein (g/day)	4.3 (2.6–7.5)
Urinary protein ≥3.5 (g/day) [*n* (%)]	114 (66.7)
Total cholesterol (mg/dL)	285 (238–372)
Leg edema [*n* (%)]	131 (76.6)
Pleural effusion [*n* (%)]	33 (19.3)
ACE inhibitor or ARB therapy [*n* (%)]	156 (91.2)
Initial immunosuppressive therapy [*n* (%)]	101 (59.0)
Prednisolone [*n* (%)]	30 (17.5)
Prednisolone + cyclosporine [*n* (%)]	55 (32.1)
Interval until start of immunosuppressive therapy (months)	0.7 (0–24)
Observation period (months)	37 (16–71)
Outcomes	
Remission	
Complete remission (CR) [*n* (%)]	103 (60.2)
Incomplete remission I (ICR I) [*n* (%)]	27 (15.8)
Incomplete remission II (ICR II) [*n* (%)]	20 (11.7)
No remission (NR) [*n* (%)]	21 (12.2)
Relapse [*n* (%)]	26 (15.2)
30% reduction in eGFR [*n* (%)]	37 (21.6)
Decline in eGFR (mL/min per 1.73m^2^ per year)	2.5 (0–8.3)
ESRD [*n* (%)]	2 (1.1)
Death [*n* (%)]	11 (6.4)
Death due to infection [*n* (%)]	7 (4.1)
Hospitalization due to infection [*n* (%)]	13 (7.6)
Hospitalization due to cardiovascular disease [*n* (%)]	3 (1.8)
Malignancy [*n* (%)]	5 (2.9)

Median (interquartile range), Conversion factors for units: SCr in mg/dL to μmol/L, × 88.4; eGFR (mL/min/1.73 m^2^) = 194 × Scr^−1.094^ × Age^−0.287^ × 0.739 (if female), total cholesterol in mg/dL to mmol/L, × 0.02586.

IMN: idiopathic membranous nephropathy; eGFR: estimated glomerular filtration rate; ACE inhibitor/ARB: angiotensin-converting enzyme inhibitor/angiotensin receptor blocker; SCr: serum creatinine; ESRD: end-stage renal disease.

### Treatment during the observation period

The enrolled patients were divided into three groups according to their treatment within 6 months of kidney biopsy: (1) the supportive therapy group, comprising 86 patients (50.3%) who received neither prednisolone nor other immunosuppressive drugs; (2) the prednisolone monotherapy group, comprising 30 patients (17.5%) who received prednisolone alone; and (3) the cyclosporine combined group, comprising 55 patients (32.1%) who received prednisolone and cyclosporine. One patient in the cyclosporine group (0.6%) also received mizoribine. The time from renal biopsy to the initiation of immunosuppressive therapy was 0.7 months (interquartile range, 0.3–3.3 months). By the end of follow-up, 156 (91.2%) patients used an ACE inhibitor or ARB.

### Outcome data

**Figure 1.**
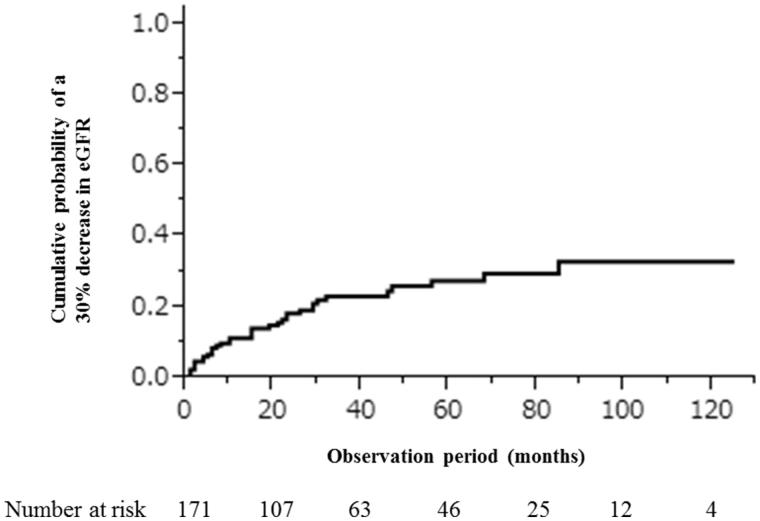
Cumulative probability of developing a 30% decrease in eGFR.

During the median, 37 months (interquartile range, 15–72 months) of observation for the cohort (*n* = 171), 37 (21.6%) patients developed a 30% decline in eGFR before ESRD, and 2 (1.2%) progressed to ESRD. The rates of developing a 30% decline in eGFR in 1, 5, or 10 years following baseline were 0.11 (95% confidence interval [CI], 0.07–0.17), 0.28 (95%CI, 0.20–0.37), and 0.33 (95%CI, 0.23–0.44), respectively (Figure 1).

**Figure 2.**
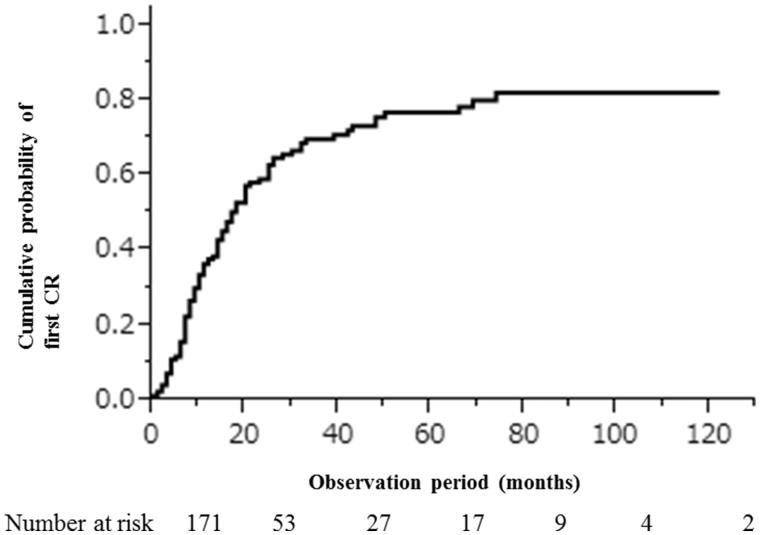
Cumulative probability of first complete remission.

Meanwhile, a total of 103 (60.2%) patients achieved CR and 27 (15.8%) patients had ICR I, 20 (11.7%) patients had ICR II, and 21 (12.2%) patients had persistent nephrotic syndrome. The cumulative probabilities of first CR within 1, 5, and 10 years were 0.38 (95%CI, 0.30–0.46), 0.77 (95%CI, 0.68–0.84), and 0.82 (95%CI, 0.72–0.89), respectively (Figure 2). Among the patients who achieved a first remission, 26 (15.2%) relapsed at least once. Infection caused 13 (7.6%) individual patient hospitalizations and 7 (4.1%) deaths in mostly elderly patients (≥65 years), as previously reported [9].

Of the total 11 (6.4%) patients who died during the study period, the remaining causes of death were acute subdural hematoma (*n* = 1), traffic accident (*n* = 1), sudden death (*n* = 1), and intestinal bleeding (*n* = 1). Malignancy occurred in five (2.9%) patients, whose diagnoses included esophageal cancer (*n* = 1), stomach cancer (*n* = 1), colon cancer (*n* = 1), prostate cancer (*n* = 1), and malignant lymphoma (*n* = 1). Only three (1.8%) patients were hospitalized for cardiovascular disease, and no patient had a venous thromboembolic event, as previously reported [8,9].

### Predictors of a 30% decline in eGFR

Time-dependent multivariate Cox regression models revealed that a lower level of proteinuria over time was significantly associated with a lower risk of a 30% decrease in eGFR (primary outcome), adjusting for clinically relevant factors (Table 2). CR (adjusted hazard ratio (HR), 0.005 [95%CI, 0.00–0.08], *p* < .001), ICR I (adjusted HR, 0.01 [95%CI, 0.001–0.19], *p* = .002) and ICR II (adjusted HR, 0.11 [95%CI, 0.02–0.59], *p* = .010) were significantly associated with avoiding a 30% decrease in eGFR as compared to NR. These results suggested that the risk of renal progression increased almost linearly over time with the amount of proteinuria.

**Table 2.** Predictors of a 30% decline in eGFR.

	Multivariate model	
	HR (95%CI)	*p* value
Baseline data		
Age (per year)	0.99 (0.94–1.03)	.501
Male (versus female)	1.49 (0.63–3.50)	.366
Systolic blood pressure (per 1 mmHg)	1.00 (0.98–1.01)	.645
Serum albumin (per 1.0 g/dL)	1.15 (0.59–2.23)	.681
Serum creatinine (per 1.0 mg/dL)	2.00 (0.45–8.84)	.361
ACE inhibitor or ARB therapy	0.44 (0.12–1.59)	.210
Urinary protein (per 1.0 g/day)	1.13 (0.99–1.29)	.060
Follow-up data		
Urinary protein over time		
No remission (NR; ≥3.5 g/day)	Reference	
Incomplete remission II (ICR II; 1–3.5 g/day)	0.11 (0.02–0.59)	.010
Incomplete remission I (ICR I; 0.3–1g/day)	0.01 (0.001–0.19)	.002
Complete remission (CR; < 0.3g/day)	0.005 (0.0–0.08)	<.001
Immunosuppressive treatment		
No immunosuppressive agents	Reference	
Prednisolone	0.44 (0.08–2.53)	.355
Prednisolone + cyclosporine	2.45 (0.34–17.7)	.376

Data are the HR, 95%CI, and *p* values, adjusted for baseline data (age, sex, systolic blood pressure, serum creatinine level, serum albumin level, urinary protein level, and use of ACE inhibitors or ARBs within 6 months after kidney biopsy) from Cox regression models, and follow-up data (urinary protein and immunosuppressive treatment) from time-dependent Cox regression models.

‘No remission’ was used as the reference category.

IMN: idiopathic membranous nephropathy; ACE: angiotensin-converting enzyme; ARB: angiotensin receptor blocker; HR: hazard ratio; CI: confidence interval.

Furthermore, as in our previous study, we identified cigarette smoking as a key dose-dependent predictor of IMN progression (Supplementary Tables 1 and 2).

### eGFR during follow-up period and comparison of proteinuria groups

**Figure 3.**
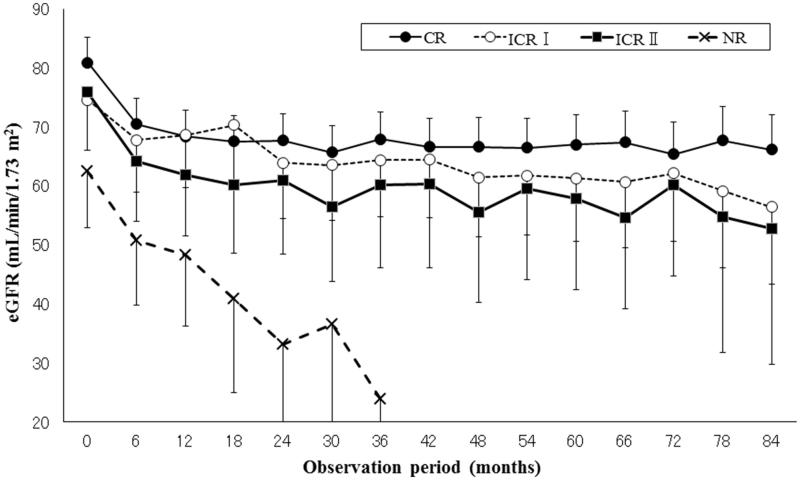
The course of eGFR during the follow-up period (comparison of the four proteinuria groups).

eGFR data during the follow-up period was compared with the level of proteinuria that each patient achieved one time (Figure 3). The eGFR in the NR group was significantly lower during the follow-up period than that in the CR, ICR I, and ICR II groups (*p* < .001).

## Discussion

We report here for the first time that exposure to proteinuria over time is an important predictor of kidney dysfunction; the lower the level of proteinuria, the better the renal prognosis, even after adjusting for immunosuppressive treatment over time using time-dependent Cox regression models. We also suggested that achieving subnephrotic proteinuria can be considered as an adequate goal.

Results of the present study were compatible with those of previous studies; however, the earlier findings [4,5] should be validated in the present study for several reasons. First, previous studies did not include data on urinary protein over time or the regimen of immunosuppressive therapy in their analyses [4,5]. These covariates are important, because, in general, proteinuria in IMN may change often, and the choice of therapeutic interventions, such as immunosuppressive agents, may change accordingly. Therefore, the analysis of the previous studies was inadequate to assess the relationship between proteinuria and renal prognosis of IMN. Second, previous studies have demonstrated clinical differences in patients with IMN across different geographic regions of the world. Population aging is now recognized as a global issue of increasing importance in many countries. In particular, the population in Japan is rapidly aging and is already one of the highest in the world. The present study also had more elderly patients than studies from other countries, as also observed among patients with IMN [1–9]. Therefore, it is important to confirm whether the findings from previous studies are valid in Japan. Third, treatment modalities differ over time. For example, renin-angiotensin system (RAS) inhibitors are considered effective conservative therapy for reducing urinary protein levels in patients with IMN, but the proportion of patients who were prescribed RAS inhibitors in a previous study was relatively lower than that in the present study [5]. This lower proportion may be owing to changes in treatment regimens over time, and therefore, the results should be interpreted carefully.

For the reasons mentioned earlier, it seemed important to confirm previously reported associations between renal prognosis and urinary protein in a Japanese population. For this purpose, we thought that the time-dependent Cox regression models, adjusting for immunosuppressive treatment over time, would be the most appropriate approach.

Similar to the previous study, the present study demonstrated that achieving lower proteinuria levels is an important predictor of renal survival in patients and is required for the stability of kidney function, suggesting that intensive therapy to reach lower levels of proteinuria is justified. However, currently used immunosuppressive treatment modalities have resulted in significant toxicity and elderly patients in our study who received immunosuppressive treatment were significantly predisposed to infection, as reported in previous studies [14–17]. Therefore, selecting patients at high risk of progression and setting an appropriate target of proteinuria are important for minimizing treatment-related adverse events. Our results provide insights on the value of proteinuria reduction that could be considered a therapeutic target, especially in elderly patients.

Our retrospective cohort study has some limitations. First, this study was not a therapeutic trial and was not designed to assess the benefit of therapeutic interventions in the course of IMN; therefore, it is unclear whether the reported associations reflect causal relationships. Although this study points a potentially important target level of proteinuria for treatment in IMN patients, residual confounding certainly remains a potential problem in such retrospective studies. Second, this study was not designed to assess the role of renal biopsy findings in predicting outcomes. Third, a 30% decline in eGFR has been confirmed as the surrogate marker of ESRD in the general population. However, the accuracy of % decline in eGFR as a marker of ESRD in diseased populations remains unclear in various diseases [11,12]. As for idiopathic membranous nephropathy, generalizability should be assessed in further studies. Fourth, in the present study, we did not measure autoantibodies to the M-type phospholipase A2 receptor (PLA2R). Further study is needed to evaluate the renal prognosis in IMN by using the information about PLA2R. In the present study, our aim was to measure factors during the course of disease, including proteinuria over time and immunosuppressive therapy, related to kidney dysfunction.

After considering these methodological issues, our study has important advantages: it is one of the largest multicenter cohorts of adults with IMN in Japan, and it is also, to the best of our knowledge, the first to describe the relationship between proteinuria over time and renal dysfunction in patients with IMN in Japan.

In summary, achieving lower proteinuria levels is an important predictor of renal survival in patients with IMN in Japan. This study quantifies the impact of proteinuria reduction in IMN and the clinical relevance of achieving subnephrotic proteinuria in this disease as a valuable prognostic indicator for both the clinician and patient.
